# Nucleocytoplasmic Shuttling of Cytoskeletal Proteins: Molecular Mechanism and Biological Significance

**DOI:** 10.1155/2012/494902

**Published:** 2011-12-20

**Authors:** Masahiro Kumeta, Shige H. Yoshimura, James Hejna, Kunio Takeyasu

**Affiliations:** Graduate School of Biostudies, Kyoto University, Yoshida Konoe-cho, Sakyo-ku, Kyoto 606-8501, Japan

## Abstract

Various nuclear functional complexes contain cytoskeletal proteins as regulatory subunits; for example, nuclear actin participates in transcriptional complexes, and actin-related proteins are integral to chromatin remodeling complexes. Nuclear complexes such as these are involved in both basal and adaptive nuclear functions. In addition to nuclear import via classical nuclear transport pathways or passive diffusion, some large cytoskeletal proteins spontaneously migrate into the nucleus in a karyopherin-independent manner. The balance of nucleocytoplasmic distribution of such proteins can be altered by several factors, such as import versus export, or capture and release by complexes. The resulting accumulation or depletion of the nuclear populations thereby enhances or attenuates their nuclear functions. We propose that such molecular dynamics constitute a form of cytoskeleton-modulated regulation of nuclear functions which is mediated by the translocation of cytoskeletal components in and out of the nucleus.

## 1. Introduction

The cytoskeleton has a well-organized and dynamically regulated subcellular architecture composed of core filaments and various accessory proteins. A variety of cellular signaling events are supported by cytoskeletal/scaffold components, which in some cases constitute structural hubs for signaling and/or in other cases contribute directly or indirectly to signal transduction. Such roles of cytoskeletal elements are not limited to purely cytoplasmic events but extend to signals ultimately targeted to the nucleus.

Nuclear signaling is fundamentally important for cells to properly orchestrate adaptive responses to various environmental changes and functional requirements. The spatio-temporal steps involved in transducing a signal from the plasma membrane to the nucleus occur in different subcellular locations: signal reception/transduction at the plasma membrane, signal amplification and cascade in the cytoplasm, shuttling/signaling through the nuclear pore complex (NPC) at the nuclear envelope, and modulation of nuclear functions. Cytoskeletal involvement in signal transduction is well documented in signal recognition at the plasma membrane and the signal cascade in the cytoplasm. Many membrane receptors and transporters interact with the cytoskeleton (e.g., ankyrin and spectrin) and transmit their specific signals to their respective target sites. Thus, the cytoskeleton often functions as a platform for signal transduction in the cytoplasm and has been assumed to only indirectly contribute to the subsequent steps of nuclear signaling, that is, signal transmission to the nucleus and regulation of nuclear functions.

There is increasing evidence, however, that many types of cytoskeletal proteins are localized to the nucleus, suggestive of their direct involvement in the transmission of nuclear signaling and the regulation of nuclear functions (Table 1). Some of these are predominantly localized to the cytoplasmic side and nearly undetectable in the nucleus by using fluorescence techniques under normal conditions; even so, a small subpopulation of this class of proteins shuttles between the cytoplasm and the nucleoplasm [1, 2]. In another case, homologues, variants or fragments of several cytoskeletal proteins are predominantly found in the nucleus [3, 4]. Such cytoskeletal proteins are strong candidates for components of the nucleoskeleton, the structural basis for nuclear functions. In addition to the LINC complex, which links the cytoskeleton and nucleoskeleton by SUN-KASH protein interactions penetrating through the nuclear membrane [5], the two compartments may be directly connected via the exchange of structural molecules. To fully understand the biological significance of the cytoskeletal proteins in the nucleus, the molecular mechanisms of their nuclear translocation should be clarified. We will review the studies of cytoskeletal proteins in the nucleus and propose possible mechanisms that regulate their nuclear localization.

## 2. The Role of Cytoskeletal Proteins in the Nucleus

Actin is well known as a component of actin filaments and is one of the most abundant proteins in cells. The first report of actin in the nucleus was published in 1969 [32], and the association of nuclear actin with RNA polymerase II was found subsequently [33–35]. A report in 1998 revealed the nuclear export signal- (NES-) dependent active nuclear export of actin through the NPC [1]. Thereafter, multiple roles of nuclear actin have been revealed, including transcription [36–39], chromatin remodeling [40], and mRNA transport [41, 42]. Furthermore, varieties of other cytoskeletal proteins are localized to the nucleus and are involved in the regulation of nuclear functions (Table 1). The notion that localization of cytoskeletal proteins in the nucleus is indispensable for proper nuclear functions is now gaining widespread acceptance.

### 2.1. Nuclear Actin, Myosins, and Associated Proteins

Nuclear actin has been implicated in a variety of nuclear functions, such as transcription [36–39], chromatin remodeling [40], and molecular transport of mRNA [41, 42] and proteins [43]. It associates with an SWI/SNF-like chromatin remodeling complex [44], hnRNPs [39, 42, 45], and RNA polymerases I, II, and III [46–50]. These findings of nuclear actin in various fundamental biological processes substantiate the constitutive requirement for actin in the nucleus. One of the most famous actin-interacting proteins in the cytoplasm, myosin, is also found in the nucleus. Nuclear myosin I (NMI) is a monomeric and single-headed myosin, which possesses an additional N-terminal 16 amino-acid domain that directs the molecule into the nucleus [7, 8]. Unlike nuclear actin, NMI is a nuclear-specific isoform, which is almost exclusively localized to the nucleus. NMI functions cooperatively with nuclear actin and especially participates in the association with the chromatin remodeling complex and RNA polymerases [47, 51].

The precise molecular states of actin in the nucleus, the monomeric or polymeric form, have not yet been fully resolved. A role of nuclear actin in the serum-induced gene activation pathway favors monomeric actin in the nucleus. MAL, a coactivator of the serum response transcription factor (SRF), is known to interact with monomeric actin [52]. Under normal conditions, MAL-actin complexes are actively exported from the nucleus, possibly due to the NES of the actin molecule. When serum-induced signaling is triggered, the MAL-actin interaction is disrupted, resulting in the accumulation of MAL in the nucleus and the activation of SRF target genes [53]. On the other hand, polymerized actin is required for the transcription of ribosomal RNA by RNA polymerase I [50]. Nuclear actin and NMI cooperate in the activation of rRNA transcription, and actin-dependent motor activity of NMI is required for transcription elongation. Coupled with the observation of actin-containing filamentous structures around the NPC [54], it is likely that nuclear actin exists in polymerized states, at least to some extent. Considering the cellular concentration of actin, which is estimated to be ≤100 **μ**M, remarkably higher than the critical concentration for polymerization [55], and also the high abundance of actin in *Xenopus* oocyte nuclei [56], it is reasonable to assume that both monomeric and polymeric forms of actin exist in the nucleus. Mobility measurements of nuclear actin using fluorescence recovery after photobleaching (FRAP) provided important supporting evidence. Recovery of the fluorescence of GFP-*β*-actin demonstrated that nuclear actin contains several different kinetic populations, suggesting that approximately 16% of the nuclear actin is in a polymeric form [57]. In a recent study demonstrating the role of actin and actin-interacting protein in *Xenopus* oocytes, polymerization of nuclear actin was necessary for the transcriptional reprogramming of the *Oct4* gene [58]. These findings suggest that dynamic conformational changes of the nuclear actin in association with its interacting partners play significant roles in various cellular events, both for general biological processes and for specific signal responses.

Several different isoforms of myosins, other than NMI, were also found to localize to the nucleus. Myosin II (smooth muscle myosin) is localized to the nucleus in smooth muscle cells and regulates transcription by binding to the promoter regions of target genes and interacting with the RNA polymerase II complex [9]. Myosin VI is also detected in the RNA polymerase II complex, and it enhances transcription much like myosin II [12]. Myosin Va and Vb were recently found in nuclear speckles and nucleoli, and are involved in transcription by RNA polymerase I [10, 11]. One of the cytoplasmic actin-depolymerizing factors cofilin-1 was also found to function in the nucleus [13]. Cofilin-1 associates with a complex containing nuclear actin and phosphorylated RNA polymerase II, and it plays a key role in transcriptional elongation, presumably by regulating polymerization states of nuclear actin along target genes. Several different mechanisms were found to target Neuronal Wiskott-Aldrich syndrome protein (N-WASP), a primary cytoplasmic regulator for cortical actin filaments, to the nucleus. N-WASP contains both a nuclear localization signal (NLS) and NES. N-WASP phosphorylated by focal adhesion kinase at a tyrosine residue Y256 is predominantly localized to cytoplasm while wild-type and kinase-insensitive mutant Y256F are localized both in cytoplasm and in the nucleus [14]. Coprecipitation assays demonstrated that N-WASP interacts with a multifunctional large nuclear protein complex, PSF-NonO (polypyrimidine-tract-binding-protein-associated splicing factor-non-Pou-domain octamer-binding protein/p54nrb), that functions to couple N-WASP with RNA polymerase II to regulate transcription [15]. Since N-WASP modulates actin polymerization in nuclear extracts *in vitro*, it is suggested that nuclear N-WASP promotes polymerization of nuclear actin to regulate transcription. It is likely that the regulation of actin polymerization is a critical factor for nuclear transcription, and there are various factors that cooperatively modulate the process, just as in the cytoplasm.

### 2.2. Actin-Related Proteins

There are at least ten actin family proteins that are conserved among eukaryotes, termed actin-related proteins (Arps), which show 30–70% similarity with actin and possess a similar ATP binding motif [59]. In spite of their well-conserved molecular structures, each Arp exhibits a distinctive role and distribution in cells. Six of them, including Arp4, 5, 6, 7, 8, and 9, displayed predominant nuclear localization in *S. cerevisiae*, and it is also the case for at least four human Arps, Arp4, 5, 6, and 8 [3, 16]. In addition to nuclear actin, these nuclear Arps are known to serve as a functional subunit of evolutionally conserved chromatin remodeling complexes, such as INO80, SWR1, RSC, and ADCR [60–62]. Taking into account the ATP-dependent mechanical sliding or displacement of nucleosomes by a chromatin remodeling complex, nuclear actin and Arps may function as a structural element for chromatin dynamics, just as the cytoplasmic actin cytoskeleton serves as a platform for various molecular dynamics.

Several nuclear Arps are known to participate in chromosomal organization and mitotic chromosomal segregation without associating with chromatin remodeling complexes. Arp6 binds to several chromatin regions including centromeres and the promoters of ribosomal protein genes [63]. This association is required to anchor a specific chromatin region to the nuclear periphery, suggesting a role of Arp6 in organizing proper chromatin regions in the nucleus. Arp8 is targeted to mitotic chromosomes and plays a unique role in chromosome segregation. When the endogenous Arp8 was depleted by siRNA transfection, mitotic cells exhibited abnormal chromosome alignment [64]. Some of the Arps are expressed in a tissue-specific manner [65]. Human ArpT1 and mouse ArpM1 are exclusively expressed in testis [66, 67]. ArpM1 interacts with profilin III, a testis-specific nucleotide exchange factor for actin, suggesting that the ArpM1-profilin complex functions in spermiogenesis-specific chromosomal organization. It is expected that nuclear Arps may participate in specific steps of development or differentiation, while widely expressed nuclear actin is involved in the regulation of general nuclear functions.

### 2.3. Spectrin-Repeat Proteins

The spectrin-repeat (SR) is a widely conserved domain from bacteria to humans, found in several cytoskeletal crosslinking proteins such as spectrin, actinin, dystrophin, and nesprin. A single SR is a rod-shaped structure consisting of a bundle of three amphiphilic *α*-helices facing each other via hydrophobic interactions, forming a hydrophobic inner surface with a hydrophilic outer surface [68]. SR-containing cytoskeletal crosslinkers possess multiple SRs and crosslink their target filaments into variously arranged forms, such as a contractile bundle, parallel array, or meshwork. In the last few years, several SR proteins have been found in the nucleus [69].

*α*-Actinin contains four SRs at the center of the molecule and forms a homodimer to crosslink actin filaments. One of the nonmuscle *α*-actinin isoforms, actinin-4, was found to be localized to the nucleus in some cancer cell lines [17]. Actinin-4 does not contain an NLS and possesses at least one functional NES, which regulates the nucleocytoplasmic shuttling of this molecule in a CRM1-dependent manner [2]. Nuclear actinin-4 is coprecipitated with the INO80 chromatin remodeling complex, which contains nuclear actin and several Arps, and is involved in cell cycle-dependent gene expression. It has also been revealed that a splicing variant of actinin-4 that lacks the amino acids 89–478 is predominantly localized to the nucleus and mediates the transcription of the *TAF55* gene in association with myocite enhancer factor-2 [70]. It is likely that actinin-4 maintains cooperative relationships with actin in the nucleus, just as in the cytoplasm, though the detailed mechanisms of the molecular interaction have not yet been fully revealed.

Spectrins are also known to function in the nucleus. Spectrins are large proteins (over 200 kDa) containing a tandem array of sixteen to twenty SRs. As a crosslinker of the actin cytoskeleton in the cytoplasm, spectrins form a tetramer consisting of two each of *α*- and *β*-spectrins. It has been reported that an isoform of nonerythroid *α*-spectrin (*α*IISp) is required for recruitment of the DNA-repair proteins FANCA and XPF to the sites of DNA interstrand crosslinks [18]. The *α*IISp isoform from patients of Fanconi anemia exhibits reduced stability, which correlates with a decreased level of DNA repair activity [19]. This deficiency results in mitotic chromosomal aberrations, suggesting a role of nuclear *α*IISp as a scaffold for DNA repair. One of the truncated isoforms of *β*-spectrin, *β*IVSpΣ5 (72 kDa), is localized to the promyelocytic leukemia (PML) bodies [4]. This protein is tightly bound to the highly insoluble nuclear scaffold and overexpression of GFP-*β*IVSpΣ5 results in an increase in the number of PML bodies, suggesting that spectrin *β*IV may be involved in the genesis of PML bodies. Interestingly, a screen for proteins that co-immunopreciptated with *α*IISp identified several plausible candidate interacting proteins, including *β*IVSpΣ5, actin, lamin A, emerin, and PML [71]. In addition, the screen identified proteins associated with DNA repair (e.g., Rad51, Rad50, Ku70, Ku80, FANCA, FANCG, FANCD2, XPF, XPG, RPA70), chromatin remodeling (e.g., BRG1, BRM), and RNA processing (hnRNP A2/B1, ribosomal-associated protein p40). Many of these interacting proteins associate in their own complexes; so it is not surprising that immunoprecipitation of *α*IISp pulled down so many candidates. The results are consistent with a role of nuclear spectrins as dynamic scaffolds for coordinating diverse nuclear complexes [18].

Several other SR proteins such as nesprins and BPAG1 have also been found in the nucleus [20–22]. These proteins are expected to play a variety of roles in the nucleus, such as organization of nuclear lamina [72] and regulation of MAPK signaling by tethering ERK1/2 at PML bodies [73]. Since most of these SR proteins contain actin binding domains, nuclear SR proteins may have cooperative functions with nuclear actin and Arps, possibly serving as a structural base or platform for their functions.

### 2.4. Cell Adhesion Proteins

*β*-Catenin, one of the cell adhesion molecules, is also known to be a signal transduction molecule for the canonical Wnt signaling pathway. Wnt signaling is one of the key signaling pathways in embryonic development and other processes, including bone formation and homeostasis, as well as adult tissue maintenance; dysregulation of Wnt signaling is implicated in some cancer and degenerative diseases [23, 74–76]. The canonical Wnt pathway regulates cell fate by modulating gene expression directed by the nuclear accumulation of *β*-catenin [77]. As the number of reports on Wnt signaling is rapidly increasing, we will mainly focus on the molecular interactions related to the nuclear translocation of *β*-catenin.

The primary subcellular localization of *β*-catenin is in the adherence junctions. At the junction sites, *β*-catenin is bound to E-cadherin and *α*-catenin and functions to link the actin cytoskeleton to the plasma membrane [78]. The adherence junction complex is dynamically regulated by the association of actin, *α*- and *β*-catenins, cadherin, and also actin-bundling proteins such as actinin and vinculin, which was demonstrated *in vitro* and on isolated cadherin-containing membrane patches [79]. At the resting stage, some of the *β*-catenin molecules escape from the cytoskeletal organization to the cytoplasm. These free *β*-catenins are captured by adenomatous polyposis coli (APC) and are phosphorylated by glycogen synthase kinase 3*β* (GSK3*β*) and then ubiquitinated and consigned to proteasomal degradation [74]. When the canonical Wnt pathway is activated by the binding of Wnt to the membrane receptor, this degradation pathway is inactivated due to the inhibition of *β*-catenin phosphorylation. This results in a high level of the free molecules in the cytoplasm and leads to their nuclear accumulation [76]. In the nucleus, *β*-catenin interacts with the transcription factor LEF-1, which activates transcription of the target genes [24]. Thus, the key event in Wnt signal transduction is the molecular transport of *β*-catenin from the cytoskeleton to the nucleus. This finding is a remarkable example of cytoskeleton-modulated regulation of nuclear functions, which is mediated by the direct translocation of a cytoskeletal component into the nucleus.

Zyxin, a 61 kDa integrin-associated focal adhesion protein, possesses a functional NES and shuttles between the cytoplasm and the nucleoplasm [25, 80]. The nuclear translocation of zyxin is triggered by cGMP. Zyxin promotes nuclear translocation of active Akt and participates in antiapoptotic cell survival of cardiomyocytes [81]. Zyxin is also translocated into the nucleus in response to mechanical stress and regulates mechanosensitive gene expression [82]. One of the zyxin-family proteins Lipoma-Preferred Partner (LPP), which is predominantly localized to cell adhesion sites and interacts with the tumor suppressor protein Scrib, was shown to shuttle into the nucleus in a similar fashion as zyxin [26]. LPP interacts with, and functions as a coactivator of, the transcription factor PEA3, which plays important roles in development and oncogenesis [27].

### 2.5. Other Cytoskeletal Components

While many reports describe nuclear localization and functions of actin-related cytoskeletal proteins, several other types of cytoskeletal proteins may also shuttle into the nucleus. In many organisms, including yeast and most fungi, the nuclear membrane does not disassemble during mitosis. In yeast, the *γ*-tubulin homologue Tub4p is actively imported into the nucleus to form a spindle pole body on the nucleoplasmic side [83]. This nuclear transport is mediated by Spc98p, an NLS-containing component of the yeast *γ*-tubulin complex. A study on fungi has demonstrated that tubulin is excluded from interphase nuclei but is present in mitotic nuclei [84]. Live observation of GFP-coupled tubulin revealed that tubulin enters the nucleus seconds before the formation of the mitotic spindle and is removed from the nucleus at the M-to-G1 transition. Nuclear tubulins are also found in several types of higher eukaryotic cells [85]. Immunofluorescence analyses of several normal and cancer-derived human cells have revealed *β*II-tubulin, but not other isotypes, in the nucleoplasm as well as the cytoplasm only in the cancer cell lines [28]. *β*II-Tubulin accumulates in the nucleus after taxol treatment, and it associates with the Notch 1 receptor intracellular domain (N1IC), the activated form of Notch 1 receptor [86]. It has also been reported that nuclear *γ*-tubulin coprecipitated with Rad51 and colocalized with Rad51 in DNA repair foci after treatments with various DNA-damaging reagents [29]. Though it has not been fully revealed whether nuclear tubulins are a general phenomenon for normal cells, these findings suggest roles for nuclear tubulins in various nuclear events.

Vimentin, one of the building blocks of type-III intermediate filaments (IFs), has also been found in the nucleus. Vimentin directly interacts with DNA through its Arg-rich N-terminal head domain [30]. Interestingly, coinjection of FITC-labeled vimentin with various single-stranded oligodeoxyribonucleotides or double-stranded circular DNA resulted in the nuclear accumulation of fluorescently labeled vimentin [31]. This N-terminal DNA binding domain is responsible for the morphological changes of the nucleus induced by the human immunodeficiency virus (HIV) type-1 protease, suggestive of the role of nuclear vimentin as a scaffold for chromatin organization [87]. More recently it was reported that vimentin upregulates the expression of p21Waf1, a cyclin-dependent kinase inhibitor in neuroblastoma [88], and inhibits osteocalcin gene expression in association with activating transcription factor-4 (ATF4) in osteoblasts [89]. Thus, the direct DNA binding property of vimentin suggests that its role in chromatin organization extends to transcriptional regulation.

There is also emerging evidence that other cytoplasmic IF proteins, such as keratins, localize to the nucleus [90]. Considering their conserved molecular structure and polymerization mechanisms, it is conceivable that not only vimentin but also various IF components and their accessory proteins can pass through the NPC. Cytoplasmic IFs are known to cooperatively function to provide mechanical strength to the cells, raising the intriguing possibility that those IF factors in the nucleus interact and cooperatively function with nuclear lamins, the nuclear-specific IF.

## 3. Molecular Mechanisms of Nuclear Transport

The nucleoplasm is separated from the cytoplasm by the nuclear envelope, which harbors the NPC as a selective channel/barrier for macromolecules. The NPC is an octameric structure composed of ~30 different subunits termed nucleoporins (Nups). The Nups facing the inner surface of the pore are rich in hydrophobic phenylalanine-glycine repeat motifs (FG-Nups) that are expected to form a meshwork of hydrophobic barriers inside the pore [91, 92]. Water, ions, and molecules smaller than ~40 kDa seem to pass through the pore by passive diffusion [93]. Transportation of larger proteins often requires the help of karyopherins, a family of transport mediators [94]. Karyopherins interact with their specific cargos to be transported; for example, the importin *α* and *β* complex recognizes and imports NLS-containing proteins, and CRM1 exports NES-containing proteins [95]. These two pathways, passive diffusion and karyopherin-mediated transport, are considered to be the two major mechanisms for molecular transport through the NPC.

Nuclear transport serves as a critical regulatory step for signal transduction from the cytoplasm to the nucleus in some cases. For example, NF-*κ*B, a transcriptional factor involved in multiple signaling events related to cell differentiation, apoptosis, and immune responses, is rapidly imported into the nucleus in response to various stimuli. In the TNF*α*-stimulated transport of NF-*κ*B p50/p65 heterodimer into the nucleus, importin *α* recognizes the NF-*κ*B NLS only when NF-*κ*B is released from its negative regulatory binding partner, I*κ*B*α* [96]. In another case, phosphorylation of the Ser385 residue of Epstein-Barr virus nuclear antigen 1 (EBNA-1) upregulates the NLS-dependent nuclear import of this molecule, while phosphorylation of Ser386 and Ser383 downregulates it [97]. These findings illustrate diverse ways with which the nuclear transport of functional molecules is precisely regulated.

**Figure 1:**
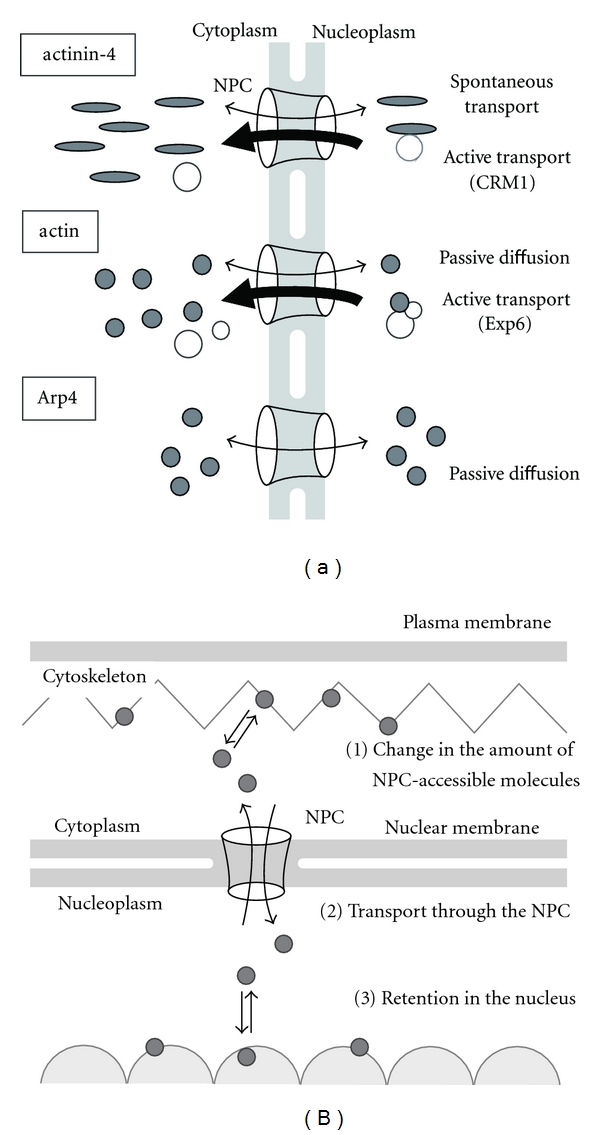
Regulatory mechanisms of cytoskeletal proteins in the nucleus. (a) Possible molecular mechanisms of the nuclear shuttling of actinin-4, actin, and Arp4 through the NPC. Actinin-4 and actin are predominantly localized to the cytoskeleton whereas Arp4 is a nuclear-specific actin homologue. In addition to the bidirectional passage by passive diffusion or spontaneous transport, actinin-4 and actin are actively exported by karyopherins, leading to their predominant localization in the cytoplasm. Arp4 does not contain functional NLS or NES in its amino acid sequence, suggesting that passive diffusion is a driving force for the nuclear transport of this molecule. It may be possible that their transport through the NPC is effected by unknown karyopherins or their binding partners, especially in the case that they possess signal sequences such as NLS or NES. (b) Three possible steps regulating the nuclear localization of cytoskeletal proteins. The balance of nucleocytoplasmic distribution can be altered by (1) a change in the amount of NPC-accessible molecules in the cytoplasm, (2) transport through the NPC, which is regulated by the combination of passive diffusion, active transport, and karyopherin-independent spontaneous transport, and (3) retention in the nucleus.

It may also be reasonable to predict that in cells that reform the nuclear membrane upon completion of mitosis, some cytoskeletal molecules can bind to nuclear material (e.g., chromatin) during mitosis and thereby be passively localized to the interphase nucleus. This may partially contribute to the nuclear localization of some cytoskeletal proteins, especially the proteins with many nuclear interactors such as actin. However, we believe that there should be a transport pathway through the NPC to regulate their nuclear populations. Considering the case of yeast cells, in which the nuclear membrane does not break down during mitosis, and of terminally differentiated somatic cells that have exited the cell cycle, the nuclear population cannot be maintained without transport through the nuclear envelope. Though it is not proven, it is posited that nuclear transport is a significant, practical mechanism to regulate nuclear populations of cytoskeletal proteins, even for the constitutively-nuclear proteins. In the following we focus on the molecular mechanisms underlying nucleocytoplasmic shuttling of cytoskeletal proteins through the NPC and propose possible regulatory factors determining their nuclear localization (Figure 1).

### 3.1. Regulation by Transport Mediators

While it is certainly true that karyopherin-dependent active transport contributes to the nuclear localization of many nuclear proteins, the nuclear transport mechanisms of cytoskeletal proteins, in particular, have not yet been fully elucidated. Nuclear export, rather than import, is shown to play a central role for regulating the nuclear localization of several cytoskeletal proteins, such as actin, actinin-4, Arp5, and Zyxin [1, 2, 25, 98]. In some cases karyopherins do not directly recognize the molecules but indirectly interact with them through mediating factors. There is strong evidence that exportin 6 (Exp6) critically functions in the nuclear export of actin [6]. Interestingly, Exp6 recognition of actin is significantly enhanced by the formation of an actin-profilin complex, suggesting a novel role of profilin as a cofactor for the nuclear export of actin. This is also the case with the *β*-catenin-APC complex. APC itself shuttles between the cytoplasm and the nucleoplasm and enhances active export of *β*-catenin to the cytoplasm in the absence of Wnt stimulation [99]. Leucine zipper tumor suppressor 2 (LZTS2) and Ran-binding protein 3 (RanBP3) were also found to function as nuclear exporters of *β*-catenin [100, 101]. Taken together, nuclear localization of several representative cytoskeletal proteins is largely determined by controlling their nuclear export.

### 3.2. Karyopherin-Independent Nuclear Transport

There is increasing evidence of karyopherin-independent nuclear transport of several cytoskeletal proteins. Whereas it is generally accepted that molecules smaller than the size limit for passive diffusion through the NPC (conventionally around 40 kDa), such as monomeric actin and some Arps, may freely migrate into the nucleus, some of the nuclear-localizing cytoskeletal proteins are substantially larger than the size limitation and are nevertheless able to pass through the NPC in a karyopherin-independent manner. For example, *in vitro* nuclear transport assays using semipermeabilized cells demonstrated that *β*-catenin and actinin-4 can pass through the NPC in a karyopherin-independent manner [2, 102, 103]. These proteins possess an amphiphilic structural domain in common: an armadillo repeat for *β*-catenin and SR for actinin-4, which both play an essential role in their nuclear migration [104]. Although the precise mechanism of the molecular transport through the NPC is the subject of continuing debate, surface hydrophobicity, promoting interactions with hydrophobic FG-Nups, is a critical factor [105]. This was demonstrated with bovine serum albumin (BSA), a protein not normally found in the nucleus. Chemical modification of BSA using several hydrophobic moieties on the surface significantly facilitated nuclear transport of the modified protein, demonstrating the role of surface hydrophobicity in the protein's ability to traverse the NPC [106]. By inference, cytoskeletal proteins with amphiphilic domains may adapt to the hydrophilic environment inside the NPC and spontaneously migrate into the nucleus. We speculate that this spontaneous migration will be the third mode of molecular transport through the NPC, which acts through the amphiphilic property of the molecule.

### 3.3. Regulatory Mechanisms for Cytoskeletal Proteins in the Nucleus

We suggest three possible mechanisms for the nucleocytoplasmic transport of cytoskeletal proteins through the NPC: passive diffusion, karyopherin-dependent active transport, and karyopherin-independent spontaneous transport. *In vivo*, the nucleocytoplasmic distribution of a molecule is determined by the combination of these three factors (Figure 1(a)). It is reasonable to assume that passive diffusion and spontaneous transport permit bidirectional molecular translocation, and karyopherin-dependent directional transport drives the differential distribution of the molecule. For example, in the case of actinin-4, the amphiphilic SR enables bidirectional spontaneous transport of this molecule through the NPC in addition to CRM1-dependent nuclear export. This combination of two driving forces not only results in the predominant localization of actinin-4 in the cytoplasm but also allows a small population of the protein in the nucleus. It may be possible to predict similar covert nuclear localizations of proteins by focusing on the size, shape, and hydrophobicity of the molecules.

To fully understand the nuclear localization of cytoskeletal proteins, it is also important to consider their accessibility to the NPC. In light of the studies we have mentioned in this review, we propose the following regulatory steps of nucleocytoplasmic shuttling of cytoskeletal proteins: (1) change in the amount of NPC-accessible molecules in the cytoplasm, (2) translocation through the NPC, and (3) retention in the nucleus (Figure 1(b)). The first step includes the changes in the equilibrium of soluble/polymeric molecules, active release from the cytoskeleton, and the inhibition of the degradation system in the case of *β*-catenin under Wnt stimulation, which results in the increase of NPC-accessible free molecules in the cytoplasm. If the molecule can passively diffuse or spontaneously migrate through the NPC, molecules in the cytoplasm and nucleoplasm reach a dynamic equilibrium via bidirectional transport, whereas the association with karyopherins strongly influences the differentially localized steady-state concentrations. Retention or release rate of the molecule from a nuclear binding partner may serve as another factor regulating the number of NPC-accessible molecules in the nucleoplasm. These three steps can independently modulate the ratio of molecules in the nucleus versus the cytoskeleton. In the case of Arp4 in yeast, associations with nuclear factors seem to play a central role in the dominant nuclear localization of this protein [107]. Mutation in the putative NLS in Arp4 did not affect its nuclear targeting, suggesting that Arp4 can passively diffuse through the NPC, and the binding of Arp4 with nuclear proteins prevents its escape from the nucleus. If the concentration of cytoplasmic free molecules is lowered by incorporation into the newly organized cytoskeleton, for example, the result may be the loss of NPC-accessible free molecules in the cytoplasm, rebalance of molecular distribution between nucleoplasm and cytoplasm by the molecular transport through the NPC, and a subsequent decrease in the population of molecules in the nucleus. We expect that this molecular dynamics, linking cytoplasmic and nuclear functions, may be especially relevant to the regulation of nuclear functions.

## 4. Perspectives

Transmission of environmental signals to the nucleus is fundamentally important for cells in various states to properly organize a response. In contrast to the normal nuclear proteins that are consistently retained in the nucleus, transnuclear signaling molecules should dynamically change their nucleocytoplasmic localization in response to the specific signals. We expect that nuclear shuttling and reorganization of nuclear cytoskeletal proteins will emerge as central to nuclear responses to external signals, just as they are central to transduction of those cues from the plasma membrane. Here we propose a model of cytoskeleton-modulated regulation of nuclear functions that are mediated by translocation of cytoskeletal components to the nucleus through the NPC (Figure 1). We predict that the dynamic distribution of many of these proteins will depend predominantly on their association with nuclear complexes and on active export by karyopherins.

There are increasingly numerous reports of cytoskeletal proteins in the nucleus. Ultrastructural mapping by immunoelectron microscopy revealed the nuclear localization of various cytoskeletal proteins in several foci or territories and also showed somewhat different localization patterns between HeLa cells and lymphocytes [108]. Such structural analyses will broaden our understanding of the role of cytoskeletal proteins in the nucleus. At the same time, a computational approach will help to specify the criteria for their passage through the NPC, which, especially for karyopherin-independent transit, remains largely a mystery.

Nuclear localization of cytoskeletal proteins has also been reported in plants. Plant nuclei contain a certain amount of actin. Microinjection of mammalian actin into plant cells led to preferential incorporation in the nucleus [109]. Silencing of Arp4 in *Arabidopsis* results in strong pleiotropic phenotypes such as altered organization of plant organs, early flowering, delayed flower senescence, and high levels of sterility [110]. Together with the studies on yeast and mammalian cells, these findings suggest that cytoskeletal proteins are generally involved in various nuclear events in virtually all eukaryotes. Considering the roles of the bacterial actin homologue MreB in a wide variety of biological events such as cytoskeletal organization, cell shape maintenance, cell wall biosynthesis, viral DNA replication, and gene expression [111–115], it is no wonder that actin and other associated proteins play roles in both the nuclear and cytoplasmic compartments in eukaryotic cells. Furthermore, we suggest that such cytoskeletal proteins may act to functionally link the nucleoplasm and cytoplasm to organize integrated cellular activities that are required for both basal cellular events and for responses to specific environmental changes.

While the focus of this review has been on the presence and regulation of cytoskeletal proteins in the nucleus, we would like to extend the discussion to nuclear proteins in general. Since all proteins are synthesized in the cytoplasm, all nuclear proteins should pass through the NPC, at least once. The passage through the NPC seems not so strict as previously thought. Bioinformatic analysis on whole yeast proteins revealed that only about 57% of steady-state nuclear proteins are predicted to use the classical nuclear import pathway, whereas the remaining 43% may use other mechanisms to enter the nucleus [116]. It also became apparent in that analysis that 40% of cytoplasmic proteins possess NLS consensus sequences, suggesting a limited influence of the classical NLS in the nuclear localization of molecules *in vivo*. The idea of export-based regulation of nuclear proteins may extend to other nuclear proteins, and to a general concept for understanding nuclear localization of proteins. This active and selective nuclear export system may also act to remove unneeded/harmful proteins from the nucleus. In contrast to the well-known cytoplasmic ubiquitin-proteasomal system, quality control of proteins in the nucleus is not well understood. Recently, a nuclear-specific proteolytic pathway has been identified, and enzymes catalyzing the steps of the ubiquitin-proteosomal degradation in the nucleus are being characterized in both yeast and humans [117, 118]. Thus, the nuclear border may be leakier than thought, and the nucleus appears to have two options for the clearance of unwanted proteins: export or degradation.

Currently, nuclear actin and *β*-catenin are two leading examples of constitutively required and specific signal-dependent cytoskeletal molecules, respectively, that shuttle and function in the nucleus. Considering the structural and dynamic features of cytoskeletal organizations, it is plausible that various types of cytoskeletal proteins shuttle through the NPC and cooperatively function in the nucleus. It is becoming apparent that cytoskeletal proteins in the nucleus play indispensable roles in a variety of nuclear events, even though their nuclear population is quite small. We suggest that there is a need to reconsider the potential functions of apparently cytoplasmic proteins in the nucleus, which may result in the discovery of their hidden talents.

## Figures and Tables

**Table 1:** Known human cytoskeletal proteins in the nucleus.

	MW (kDa)	Primary localization	Binding partners in the nucleus	Related nuclear functions	Reference
actin	42	cytoskeleton	RNA polymerase I/ II/III, transcription factor, chromatin remodeling complex, hnRNP, nuclear lamina, NPC, and so forth	transcription, DNA repair, mRNA transport, gene reprogramming	[1, 6]

nuclear myosin	120	nucleus	RNA polymerase I/II	transcription	[7, 8]

myosin II	228	cytoplasm	RNA polymerase II	transcription	[9]

myosin Va	215	cytoplasm			[10]

myosin Vb	214	nucleus	RNA polymerase I	transcription	[11]

myosin VI	149	cytoplasm	RNA polymerase II	transcription	[12]

cofilin-1	19	cytoplasm	RNA polymerase II	transcription	[13]

N-WA5P	55	cytoplasm	RNA polymerase II	transcription	[14, 15]

Arp4	48	nucleus	chromatin remodeling complex	histone modification, transcription, DNA repair	[16]

Arp5	68	nucleus	chromatin remodeling complex	transcription, DNA repair	[16]

Arp6	46	nucleus	chromatin remodeling complex, HP1	transcription, heterochromatin formation	[16]

Arp8	71	nucleus	chromatin remodeling complex, mitotic chromosomes	transcription, DNA repair, chromosome segregation	[16]

actinin-4	105	cytoskeleton	chromatin remodeling complex	transcription	[2, 17]

*α*ll spectrin	285	cytoskeleton	FANCA, FANCG, XPF	DNA repair	[18, 19]

*β*IV spectrin *∑*5	72	nucleus	PML body	formation of PML bodies	[4]

nesprins	1011/799	nuclear envelope	lamin A/C, ERK1/2, PML body	formation of nuclear envelope, signal transduction	[20, 21]

BPAG1	650	cytoskeleton			[22]

*β*-catenin	85	cell adhesion	LEF-1	transcription	[23, 24]

zyxin	61	cell adhesion		transcription	[25]

LPP	66	cell adhesion	PEA3	transcription	[26, 27]

*β*ll-tubulin	50	cytoskeleton	N1IC	signal transduction	[28, 28]

*γ*-tubulin	51	centrosome	RAD51	DNA repair	[29]

vimentin	54	cytoskeleton	DNA (*in vitro*)	transcription, nuclear architecture	[30, 31]

## Abbreviations

Arp: Actin-related protein
NPC: Nuclear pore complex
NES: Nuclear export signal
NLS: Nuclear localization signal
NMI: Nuclear myosin I
(FG-)Nup: (Phenylalanine-glycine repeat-containing) nucleoporin
SR: Spectrin repeat.
